# The effects of visual skills training on cognitive and executive functions in stroke patients: a systematic review with meta-analysis

**DOI:** 10.1186/s12984-024-01338-5

**Published:** 2024-03-26

**Authors:** Marc Niering, Johanna Seifert

**Affiliations:** 1 Institute of Biomechanics and Neurosciences, Nordic Science, Hannover, Germany; 2 Department of Psychiatry, Social Psychiatry, and Psychotherapy, Hannover Medical School, Carl-Neuberg-Straße 1, 30625 Hannover, Germany

**Keywords:** Visual skills, Cognitive function, Executive function, Stroke rehabilitation

## Abstract

The visual system and associated skills are of particular importance in stroke rehabilitation. The process of neuroplasticity involved in restoring cognitive function during this period is mainly based on anatomical and physiological mechanisms. However, there is little evidence-based knowledge about the effects of visual skills training that could be used to improve therapeutic outcomes in cognitive rehabilitation. A computerized systematic literature search was conducted in the PubMed, Medline, and Web of Science databases from 1 January 1960 to 11 Febuary 2024. 1,787 articles were identified, of which 24 articles were used for the calculation of weighted standardized mean differences (*SMD*) after screening and eligibility verification. The findings revealed moderate effects for global cognitive function (*SMD* = 0.62) and activities of daily living (*SMD* = 0.55) as well as small effects for executive function (*SMD* = 0.20) – all in favor of the intervention group. The analyses indicate that the results may not be entirely robust, and should therefore be treated with caution when applied in practice. Visual skills training shows positive effects in improving cognitive and executive functions, especially in combination with high cognitive load and in an early phase of rehabilitation. An improvement in activities of daily living can also be observed with this type of intervention. The high heterogeneity of the studies and different treatment conditions require the identification of a relationship between certain visual skills and executive functions in future research.

**Supplementary Information:**

The online version contains supplementary material available at 10.1186/s12984-024-01338-5.

## Introduction

Stroke is one of the most prevalent neurological diseases in the world, with incidence and mortality rates decreasing by 11.3% and 34%, respectively, since 1990 [1]. A significant general risk factor is the association with low- and middle-income countries, where 87% of the disability-adjusted life years are observed, impacting individuals at a relatively young age [2]. Individual risk factors include hypertension, air pollution, diabetes, and a diet high in fat and cholesterol [3]. Primary and secondary prevention strategies have been extensively studied in the literature and illustrate a homogeneous association between risk factors and intervention or patient behavior [4, 5]. In clinical practice, treatment starts in the acute phase up to 3 weeks after stroke, where hemodynamic and metabolic factors are crucial [6]. Treatment approaches in this phase are clearly emphasized in the literature in the area of endovascular [7, 8], exosome [9, 10], and stem cell-based [11–13], as well as pharmacological [14–16] approaches. In the subacute and early chronic phase, respectively 3–11 and up to 24 weeks after stroke, cognitive rehabilitation supports the patient in regaining normal function or compensating for deficits caused by the affected brain areas [17].

McDonald et al. [18] state that there is no consensus on an approach in current stroke rehabilitation due to a lack of evidence. They emphasize the necessity for thorough investigations into the effectiveness and applicability of the many promising innovations emerging in this field. Stinear et al. [19] similarly note the persistent reliance on conventional interventions in stroke rehabilitation, highlighting, that though these are effective in increasing quality of life and activity capacity, updated strategies are needed. They see potential for novel therapies that better address the challenges of a faster-paced, multi-media world and its multiple stressors and to achieve more rapid progress, particularly in improving executive functions and motor skills. These interventions include brain-computer interfaces [20, 21], robot-assisted therapy [22], virtual-reality rehabilitation [23], and various types of physical training [24, 25], all of which can now be delivered in a goal-directed manner outside of clinical rehabilitation as home-based interventions [26]. The common element of all these interventions is that they achieve effectiveness explicitly via neuroplasticity, i.e. the adaptation of the structures and function of the brain to intrinsic and extrinsic stimuli [27]. According to Sweatt [28], especially in cognitive and executive functions, structural plasticity is implied via long-lasting, recurrent stimuli that strengthen synapses between neurons. In contrast, functional plasticity involves the strengthening of neural connections due to constant shared activity between neurons, thus adjusting the functional connectivity between brain areas. Further, neurogenesis, the brain’s ability to form new neurons as an adaptation to change, has also taken on new importance in stroke rehabilitation [29]. Guggisberg et al. [30] illustrate that the reorganization of structural network systems can compensate for impaired cognitive and motor functions due to the loss of previous pathways. They advocate for tailored sensory stimulation and the processing of complex stimuli targeting the individually affected area of the brain and its core function. Visual sequelae affect 60% of all stroke survivors [31] and are the most disabling effect following cerebral infarction due to the anatomy of the central nervous system, which is largely dedicated to vision [32].

### The role of the visual system in neuroplasticity-based stroke rehabilitation

As previously stated, current post-stroke rehabilitation approaches prioritize enhancing executive functions, recognized as pivotal for managing activities of daily living [33]. The visual system holds significant importance in shaping both structural and functional neuroplasticity, as well as neurogenesis [34–36]. Moreover, it has been specifically associated with the rehabilitation of executive functions in stroke patients [37]. According to Marinho et al. [38], there is also a mutual correlation between decision-making processes and executive functions in stroke patients, both of which affect information processing and motor action. The authors explicitly highlight the significance of sensory input quality in the decision-making process and thus for performance in executive functions. The significance of the visual system in these processes can be exemplified by everyday situations, such as crossing a green traffic light. In this scenario, many different visual stimuli affect the person, ultimately contributing to determining their behavior. Research suggests that the activity in the orbitofrontal cortex predicts successful recognition of visual objects and may receive visual input after initial sensory processing [39]. These give rise to predictions that guide specialized higher visual processing [40] and, according to the model of Borji et al. [41], fundamentally influence the decision on whether and how to respond to the traffic light. In addition to the visual stimuli, there are other cognitive stressors involved when responding to a traffic light, such as the noise level or the uncertainty about one’s walking speed. Mathews et al. [42] report that as the cognitive load increases, differences between conscious perception and the rapid, reflexive eye movements called saccades become more pronounced when individuals process situations. Since cognitive load and executive functions are negatively correlated [43–45], a bias in visual processing and thus influence on both the unconscious and conscious decision-making process is to be expected. This practical relevance of the visual system and its influence on cognitive and executive functions, as well as the transfer to activities of daily living after a stroke, serve as the objective for this paper.

The importance of investigating visual skills training in post-stroke rehabilitation is underscored by recent advances in our understanding of neuroplasticity and its role in recovery. A recent meta-analysis by Hao et al. [46] showed that the visual system plays a key role in the neuroplastic effects of virtual reality interventions in stroke patients, while Ferreira et al. [47] highlighted the importance of visual skills training during the rehabilitation process. However, visual skills-based interventions are mainly used in stroke patients for a general, interactive, and stimulating effect [48], or more recently also specifically as visual skills training for visual impairments after stroke [49]. It may be relevant for clinicians to consider whether their interventions to improve cognitive and executive function should be targeted at improving visual skills. To the authors’ knowledge, no systematic review or meta-analysis has examined the effects of visual skills training in stroke patients.

This systematic review and meta-analysis assesses the current state of research on the effects of visual skills interventions used in post-stroke rehabilitation to restore cognitive function or improve functional performance. It aims to provide relevant insights for clinical practice as well as new implications for future research.

## Methods

This meta-analysis follows the recommendations of the preferred reporting Items for Systematic Reviews and Meta-Analysis (PRISMA) statement guidelines [50], which are shown in Table S1 (Supplement 1).

### Literature search

A systematic computerized search for relevant empirical studies was performed in PubMed, Medline, EMBASE, Cochrane Library, APA PsycINFO, and Web of Science using the following Boolean search strategy: (visual OR vision OR oculomotor OR “eye movement” OR visuomotor) AND (training OR exercise OR intervention) AND (“cognitive function” OR “executive function” OR cognition OR “activities of daily living”) AND stroke. The search was limited to the following criteria: publication dates: 1 January 1960 to 11 February 2024, language: English, article type: no review. To identify further studies for the analysis, the reference lists of the included studies were subsequently screened.

### Selection criteria

To be included in the systematic review, the eligible studies had to contain relevant information regarding the PICOS (Population, Interventions, Comparators, Outcomes, Study design) approach, which is shown in Table 1. To assess the relevance, the following criteria were set: (a) Population: diagnosed stroke patients; (b) Intervention: training explicitly focused on visual skills; (c) Comparator: active or passive control group (i.e., other interventions not focused on visual skills, no training at all); (d) Outcome: at least one measure of cognitive function or activities of daily living; (e) Study design: controlled trials with pre- and post-measures. The following were set as criteria for exclusion in the selection process: (a) participants were blind or had acute eye or vision injuries (i.e., cataract); (b) inaccurate or insufficient reporting of data (i.e., no measure of central tendency and dispersion in the results section); (c) effects were examined without control condition; (d) procedures did not include measurement of parameters for cognitive function or activities of daily living; (e) cross-sectional study design or reviews. When defining the intervention criteria, special attention was paid to the fact that the included studies explicitly stated the training of visual, oculomotor, or lower or higher visual system skills as the aim of the intervention. Interventions that only use the capabilities of the visual system but do not aim to improve them, such as most virtual reality interventions, were excluded.

**Table 1:** Overview of the applied inclusion and exclusion criteria

Category	Inclusion criteria	Exclusion criteria
Population	diagnosed stroke patient	participants were blind or had acute eye or vision injuries (i.e., cataracts)
Intervention	training explicitly focused on visual skills	inaccurate or insufficient reporting of data (i.e., no measure of central tendency and dispersion in the results section)
Comparator	active or passive control group (i.e., other interventions not focused on visual skills, no training at all)	effects were examined without a control condition
Outcome	at least one measure of cognitive function or activities of daily living	procedures did not include measurement of parameters for cognitive function or activities of daily living
Study design	controlled trials with pre- and post-measures	cross-sectional study design or reviews

### Data extraction and Assessment of Methodological Study Quality

The following information was extracted from the included studies: Authors, year of publication, study population and clinical condition, total sample size and sample size per group, type of visual skills intervention, cognitive or activities of daily living parameters targeted by the intervention, and pre-and post-measurements.

The variables of interest were methods for measuring cognitive function such as the Montreal Cognitive Assessment (MoCA) [51] or the Mini-Mental State Examination (MMSE) [52] for global cognitive function. Furthermore, tests for specific executive functions, such as the Wechsler Adult Intelligence Scale digit span test (WAIS-DS) [53], and procedures that measure several cognitive domains at the same time, such as the Trail Making Test (TMT) [54], were included. In this case, the results were assigned to the outcome measure to which the test can primarily be attributed. When only median and range were reported in studies [55–59], values were converted to means and standard deviations as in Wan et al. [60]. In studies where only graphs were published as results, data values were obtained using a plot digitizer.

To determine methodological study quality and minimize the risk of bias, each eligible article was assessed independently by two authors (MN, JS) according to the Scottish Intercollegiate Guidelines Network methodology checklist for randomized controlled trials [61]. The possible classifications are low quality (-), acceptable quality (+), and high quality (++). Studies classified as unacceptable (0) were rejected. The results are shown in Table S2 (Supplement 2).

### Synthesis of results

The included studies were screened for the outcome variables of interest, which resulted in this meta-analysis focusing on different outcome measures. As many different test procedures are used in the literature, the preferred and alternative measures for each outcome were presented in Table 2 to reduce the heterogeneity of the included studies.

**Table 2:** Overview of the preferred and alternative outcome by category

Category	Preferred outcome	Alternative outcome
Global cognitive function	Mini-Mental Status Examination (MMSE; *n* = 6)	Montreal Cognitive Assessment score (MoCA; *n* = 5)
Working memory function	Wechsler Adult Intelligence Scale Digit Span score (WAIS-DS; *n* = 5)	-
Visual processing speed	Trail Making Test Part A (TMT-A; *n* = 5)	-
Cognitive flexibility	Trail Making Test Part B (TMT-B; *n* = 3)	-
Selective attention	Visual continuous performance test (V-CPT; *n* = 3)	Stroop test (ST; *n* = 2)
Activities of daily living	Modified Barthel Index (MBI; *n* = 15)	-

MoCA = Montreal Cognitive Assessment; WAIS-DS = Wechsler Adult Intelligence Scale digit span score; MMSE = Mini-Mental Status Examination; MBI = Modified Barthel Index; V-CPT = Visual continuous performance test; TMT-A = Trail Making Test Part A; TMT-B = Trail Making Test Part B; ST = Stroop test

In the category of global cognitive function, the MMSE was identified as the most common test, illustrating high factorial validity [62] and low influence on the type of stroke or other comorbidities, particularly in geriatric patients [63]. In the areas of executive function, the TMT Part A and Part B were used most as measures of visual processing speed and cognitive flexibility respectively. Part A illustrates a high sensitivity in measuring visual processing speed [54], whereas Part B is more sensitive to cognitive flexibility [64]. The WAIS-DS is referred to as the best indicator of working memory ability of all subtests and demonstrates high validity as a working memory measure [53]. When considering selective attention, the Visual continuous performance test (V-CPT) has been identified as the most commonly used measure. It is often used in the assessment of stroke patients [65], although validity has not been demonstrated [66]. The Stroop task (ST) was included as an alternative outcome because, although it encompasses several executive functions, it was originally developed as a test of selective attention [67] and has recently been associated with selective-attention related hemodynamic activity [68].

Other outcomes used in the included studies that matched the categories were listed as alternative outcomes in Table 2.

In addition, the use of different treatment modalities and their combinations during an intervention was taken into account. Treatment modality was coded using the following parameters: Training weeks/sessions and session duration. In cases where the studies considered did not contain conclusive results, the authors were contacted by email [69, 70]. If the authors did not respond to the request [69, 70], the respective study was excluded from further analysis.

### Statistical analysis

To determine the effects of visual skills training on cognitive function and activity of daily living, the standardized between-subjects mean difference was calculated as *SMD* = (mean post-test in [INT] - mean post-test in [CON]) / pooled standard deviation [71]. A random-effects meta-analysis model of Review Manager version 5.4.1 was used for calculation, where *SMD* can be positive or negative. Positive *SMD* values indicate an improvement in the measured parameters in favor of the intervention group (INT), while negative values indicate an improvement in favor of the control group (CON). According to Cohen [72], 0 ≤ 0.49 for small effects, 0.50 ≤ 0.79 for moderate effects, and ≥ 0.80 for large effects are classified and interpreted accordingly. During analysis, heterogeneity (*I*^*2*^) was calculated using the formula reported by Deeks et al. [73]: *I*^2^ = (*Q* – *df* / *Q*) * 100%, where *Q* is the chi-squared statistic and *df* represents the degrees of freedom [74]. Following Deeks et al. [73], heterogeneity can be interpreted as trivial (0 ≤ 40%), moderate (30 ≤ 60%), substantial (50 ≤ 90%), or considerable (75 ≤ 100%). To account for heterogeneity in the characteristics of the study samples, sensitivity analyses were performed by successively excluding one study. In the following, a-priori sub-groups were identified on the basis of theoretical reasoning [75]. Additionally, a separate (global cognitive function/executive functions/activities of daily living) qualitative funnel plot evaluation, as well as Egger´s regression was performed to examine a potential publication bias.

## Results

### Selection of studies

**Fig. 1:**
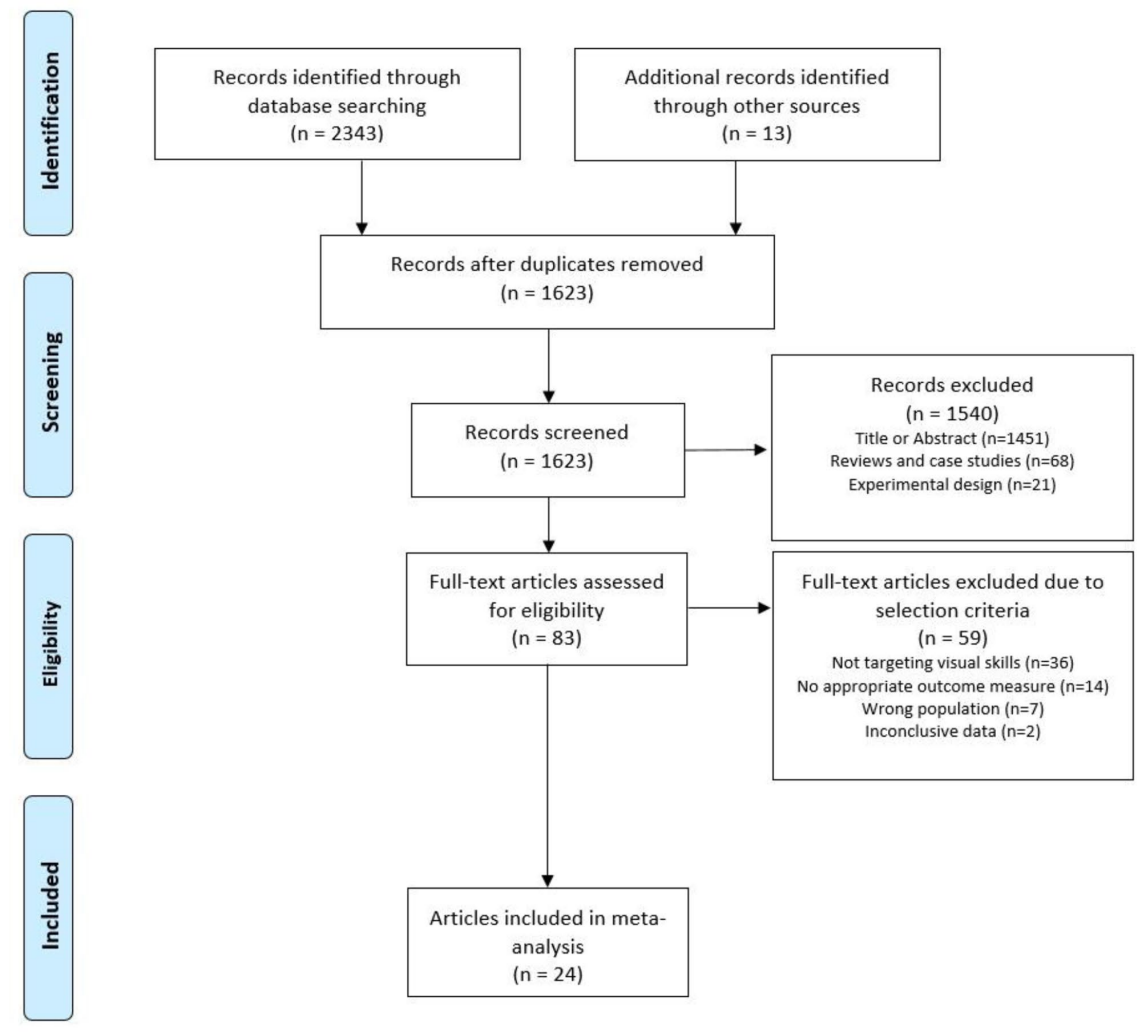
PRISMA flow diagram illustrating the systematic literature search, study selection, and respective reasons for exclusion of records

The search strategy and selection process for visual skills interventions are illustrated in Fig. 1. A total of 2,343 articles on visual skills interventions were identified for further analysis in the PubMed, Medline, EMBASE, Cochrane Library, APA PsycINFO, and Web of Science databases and supplemented by 13 additional articles derived from a manual search of the reference lists. After removing duplicates, excluding articles based on title or abstract, as well as reviews, case studies, and experimental study designs, 83 articles remained for full-text consideration. 36 articles were excluded that did not specifically focus their intervention on visual skills. Fourteen studies did not examine outcome measures relevant to the present systematic review and meta-analysis, seven did not specify only stroke patients as the population, and two did not provide conclusive data.

### Study characteristics

The characteristics of the included studies are listed in Table 3 and illustrate the authors, year of publication, participant characteristics, time since stroke, intervention and control groups, details of the interventions implemented, test procedures and outcome measures, the results of each group, and the methodological study quality.

**Table 3:** Included studies examining the effects of visual skills training on cognitive functions and activities of daily living in stroke patients

References	No. of subjects (sex); age (mean ± SD, or range); location; main symptoms	Time since stroke, mean (SD)	Groups; type of intervention; visual skills targeted	Intervention: no. of training weeks/sessions; single session duration	Outcome measures of cognitive function	Results, mean (SD)	Level of evidence
Chen et al. [55]	80 (28 F, 52 M); 57.74 ± 8.54 yrs; various; cognitive dysfunction	< 3 months	INT (*n* = 40); visual skills training CON (*n* = 40); conventional physiotherapy and gait rehabilitation	INT: 4 wk / 16 sessions; 30 min Visual skills training focused on multiple executive functions; attention, memory ability, spatial orientation CON: no details reported	MoCA (score) Global cognitive function pre-/post-intervention	**MoCA**: INT pre: 13.0 (2.2) CON pre: 13.0 (2.2) INT post: 18.0 (1.5) CON post: 14.5 (2.2)	+
Fadle et al. [76]	INT (2 F, 13 M); 54 ± 6.5 yrs; NA; NA CON (3 F, 12 M); 53.2 ± 5.91; NA; NA	INT: 8.33 (2.35) months CON: 8.66 (1.95) months	INT (*n* = 15); visuomotor training CON (*n* = 15); physical therapy and cognitive training	INT: 4 wk / 28 sessions; 30 min Visual and kinesthetic visuomotor exercises CON: 4 wk / 28 sessions; 60 min Cognitive and dual-task training, time up and go task with phonologic fluency	MoCA (score) Global cognitive function pre-/post-intervention	**MoCA**: INT pre: 21.33 (2.28) CON pre: 20.53 (1.64) INT post: 24.2 (2.54) CON post: 22.13 (1.99)	-
Schoettke [77]	INT (7 F, 9 M); 64.1 ± 8.5 yrs; various; motor dysfunction CON (7 F, 6 M); 65.4 ± 10.9 yrs; various; motor dysfunction	INT: 1.7 (0.7) months CON: 1.3 (0.4) months	INT (*n* = 16); visual-cognitive training CON (*n* = 13); physical- and occupational therapy	INT: 3 wk / 13 sessions; NA Visual-cognitive training combined with conventional rehabilitation CON: no details reported Physical- and occupational therapy	MBI (score) Activities of daily living pre-/post-intervention	**MBI**: INT pre: 69.38 (21.20) CON pre: 63.46 (26.57) INT post: 83.44 (16.90) CON post: 68.85 (26.71)	+
Pashang et al. [78]	INT (1 F, 9 M); 53.9 ± 9.73 yrs; right hemisphere; cognitive dysfunction CON (4 F, 6 M); 57.7 ± 12.16 yrs; right hemisphere; cognitive dysfunction	INT: 11.9 months CON: 20.3 months	INT (*n* = 10); visual-cognitive training CON (*n* = 10); drug- and physical therapy	INT: 8 wk / 8 sessions; 60 min Visual-cognitive training program combined with drug- and physical therapy CON: no details reported Drug- and physical therapy	V-CPT (number) Selective attention pre-/post-intervention	**V-CPT**: INT pre: 34.40 (28.63) CON pre: 55.80 (21.25) INT post: 42.70 (27.67) CON post: 58.70 (22.07)	-
He et al. [57]	INT (15 F, 17 M); 66.0 ± 6.9 yrs; various; cognitive dysfunction CON (14 F, 18 M); 67.0 ± 5.9 yrs; various; cognitive dysfunction	< 6 months	ö	INT: 6 wk / 36 sessions; 80 min 20 min of physical therapy 20 min of occupational therapy 20 min of swallowing and speech function training 20 min of eye-movement training CON: 6 wk / 36 sessions; 80 min 20 min of physical therapy 20 min of occupational therapy 20 min of swallowing and speech function training 20 min of cognitive training	MoCA (score) Global cognitive function MBI (score) Activities of daily living pre-/post-intervention	**MoCA**: INT pre: 14.6 (2.6) CON pre: 14.5 (2.4) INT post: 17.3 (2.3) CON post: 16.0 (2.3) **MBI**: INT pre: 42 (18.52) CON pre: 42 (16.30) INT post: 65 (13.33) CON post: 61 (14.07)	++
Kim et al. [79]	INT (6 F, 9 M); 70.7 ± 6.6 yrs; NA; cognitive dysfunction CON (7 F, 8 M); 71.4 ± 5.2 yrs; NA; cognitive dysfunction	INT: 4.4 (4.0) months CON: 4.9 (6.3) months	INT (*n* = 15); visuomotor training CON (*n* = 15); occupational therapy	INT: 4 wk / 32 sessions; 30 min 20 sessions of occupational therapy 12 sessions of visuomotor training CON: 4 wk / 20 sessions; 30 min occupational therapy	MMSE (score) Global cognitive function MBI (score) Activities of daily living V-CPT (number) Selective attention TMT-A (sec) Visual processing speed WAIS-DS (score) Working memory function pre-/post-intervention	**MMSE**: INT pre: 19.7 (5.6) CON pre: 19.3 (5.3) INT post: 21.1 (5.9) CON post: 22.7 (4.7) **MBI**: INT pre: 33.2 (13.7) CON pre: 46.9 (14.4) INT post: 48.7 (12.9) CON post: 61.6 (17.0) **V-CPT**: INT pre: 76.7 (31.4) CON pre: 106.4 (22.6) INT post: 100.9 (34.0) CON post: 109.2 (30.9) **TMT-A**: INT pre: 248.8 (227.2) CON pre: 283.6 (193.6) INT post: 226.9 (216.1) CON post: 273.7 (216.2) **WAIS-DS**: INT pre: 3.4 (0.5) CON pre: 3.5 (0.6) INT post: 3.3 (0.6) CON post: 3.7 (0.8)	-
Batool et al. [80]	INT (15 F, 17 M); 55.63 ± 5.90 yrs; NA; visual disorders CON (13 F, 19 M); 54.38 ± 8.78 yrs; NA; visual disorders	3–6 months	INT (*n* = 32); visual scanning training CON (*n* = 32); functional task training	INT: 4 wk / 24 sessions; 45 min 30-minute functional task training 15 min of visual scanning training CON: 4 wk / 24 sessions; 45 min 30-minute functional task training 15 min placebo random eye movements	MBI (score) Activities of daily living pre-/post-intervention	**MBI**: INT pre: 18.28 (7.47) CON pre: 20.31 (7.72) INT post: 32.66 (12.69) CON post: 26.25 (10.70)	++
De Luca et al. [56]	INT (6 F, 9 M); 30.93 ± 11.10 yrs; NA; cognitive dysfunction CON (10 F, 10 M); 39.75 ± 15.43 yrs; NA; cognitive dysfunction	3–6 months	INT (*n* = 15); visual skills training CON (*n* = 20); standard cognitive rehabilitation	INT: 8 wk / 24 sessions; NA Visual skills-based neurocognitive training combined with standard cognitive rehabilitation CON: no details reported Standard cognitive rehabilitation	MMSE (score) Global cognitive function MBI (score) Activities of daily living pre-/post-intervention	**MMSE**: INT pre: 21.2 (2.74) CON pre: 23.0 (4.81) INT post: 23.7 (2.66) CON post: 23.0 (4.22) **MBI**: INT pre: 20.0 (38.88) CON pre: 25.0 (31.48) INT post: 70.0 (29.63) CON post: 40.0 (32.5)	-
Kerkhoff et al. [81]	INT (4 F, 8 M); 64 ± 3 yrs; right hemisphere; visual disorders CON (5 F, 7 M); 64 ± 3 yrs; right hemisphere; visual disorders	INT: 1.0 (0.1) months CON: 1.2 (0.2) months	INT (*n* = 6); visual scanning training CON (*n* = 6); standard cognitive rehabilitation combined with physical therapy	INT: 4 wk / 20 sessions; 30 min 30-minute functional task training 15 min of visual scanning training CON: 4 wk / 20 sessions; 30 min 30-minute functional task training 15 min placebo random eye movements	MBI (score) Activities of daily living pre-/post-intervention	**MBI**: INT pre: 11.0 (4.0) CON pre: 15.0 (5.0) INT post: 28.0 (5.0) CON post: 26.0 (8.0)	+
Westerberg et al. [82]	INT (1 F, 8 M); 55 ± 8 yrs; various; cognitive dysfunction CON (5 F, 4 M); 53.6 ± 8 yrs; various; cognitive dysfunction	INT: 19.3 (6.2) months CON: 20.8 (6.2) months	INT (*n* = 9); visual-spatial training CON (*n* = 9); no intervention	INT: 5 wk / 25 sessions; 40 min Software-based visual-spatial cognitive training CON: -	WAIS-DS (score) Working memory function ST (sec) Selective attention pre-/post-intervention	**WAIS-DS**: INT pre: 5.8 (1.0) CON pre: 5.7 (0.9) INT post: 7.3 (1.0) CON post: 5.7 (1.3) **ST**: INT pre: 108.0 (11) CON pre: 147.0 (54) INT post: 93.0 (19) CON post: 124.0 (48)	+
Prokopenko et al. [59]	INT (4 F, 6 M); 59.5 ± 2.5 yrs; various; cognitive dysfunction CON (1 F, 8 M); 62.55 ± 2.45 yrs; various; cognitive dysfunction	< 6 months	INT (*n* = 10); visual-spatial training CON (*n* = 9); physical therapy	INT: 10 days / 10 sessions; 30–40 min Software-based visual-spatial cognitive training CON: no details reported	MMSE (score) Global cognitive function MoCA (score) Global cognitive function pre-/post-intervention	**MMSE**: INT pre: 25 (5.93) CON pre: 25.8 (5.19) INT post: 28.2 (2.96) CON post: 26.6 (5.93) **MoCA**: INT pre: 20.6 (4.44) CON pre: 20.2 (6.66) INT post: 26.1 (2.96) CON post: 21.3 (9.63)	-
Yoo et al. [83]	INT (15 F, 8 M); 53.2 ± 8.8 yrs; NA; cognitive dysfunction CON (14 F, 9 M); 56.3 ± 7.9 yrs; NA; cognitive dysfunction	INT: 11.8 (7.5) months CON: 10.7 (6.2) months	INT (*n* = 23); visual-spatial training CON (*n* = 23); physical- and occupational therapy	INT: 5 wk / 25 sessions; 30 min Visuomotor coordination, visual impairment, spatial imagination, memory, focus, attention CON: no details reported	TMT-A (sec) Visual processing speed WAIS-DS (score) Working memory function pre-/post-intervention	**TMT-A**: INT pre: 71.62 (22.31) CON pre: 68.48 (25.54) INT post: 65.68 (24.51) CON post: 67.36 (22.50) **WAIS-DS**: INT pre: 3.62 (1.35) CON pre: 3.72 (1.23) INT post: 4.32 (1.32) CON post: 3.86 (1.41)	+
Kim et al. [84]	INT (8 F, 5 M); 58.9 ± 3.1 yrs; NA; NA CON (9 F, 4 M); 57.4 ± 3.5 yrs; NA; NA	> 6 months	INT (*n* = 13); vision control dual-task training CON (*n* = 13); unstable supporting plane dual-task balance training	INT: 5 wk / 25 sessions; 30 min Visual restriction recognition task exercises CON: 5 wk / 25 sessions; 30 min Somatosensory control balance exercises	TMT-A (sec) Visual processing speed TMT-B (sec) Cognitive flexibility ST (sec) Selective attention pre-/post-intervention	**TMT-A**: INT pre: 32.9 (3.0) CON pre: 32.0 (2.7) INT post: 30.9 (2.9) CON post: 29.8 (3.1) **TMT-B**: INT pre: 45.8 (3.0) CON pre: 45.9 (6.1) INT post: 42.2 (3.2) CON post: 41.7 (6.4) **ST**: INT pre: 44.7 (7.9) CON pre: 45.9 (6.7) INT post: 40.6 (8.2) CON post: 42.1 (5.5)	+
Mazer et al. [85]	INT (12 F, 35 M); 65.5 ± 11.4 yrs; NA; NA CON (15 F, 35 M); 66.5 ± 8.9 yrs; NA; NA	INT: 3.0 (1.7) months CON: 2.2 (0.9) months	INT (*n* = 47); visual skills training CON (*n* = 50); occupational therapy and cognitive skills training	INT: 5–10 wk / 20 sessions; 30–60 min Visual skills training targetting visual processing speed, divided- and selective visual attention CON: 5–10 wk / 20 sessions; 30–60 min Somatosensory control balance exercises	TMT-A (sec) Visual processing speed TMT-B (sec) Cognitive flexibility pre-/post-intervention	**TMT-A**: INT pre: 67.5 (3.9) CON pre: 67.7 (36.0) INT post: 49.4 (16.1) CON post: 57.3 (27.6) **TMT-B**: INT pre: 207.2 (147.0) CON pre: 207.2 (116.3) INT post: 139.9 (66.0) CON post: 161.6 (81.2)	++
Liu et al. [58]	INT (8 F, 22 M); 52.5 ± 7.5 yrs; right hemisphere; motor dysfunction CON (11 F, 19 M); 53.0 ± 14.5 yrs; right hemisphere; motor dysfunction	INT: 0.6 months CON: 0.6 months	INT (*n* = 30); visual feedback training CON (*n* = 30); functional electric stimulation combined with conventional therapy	INT: 3 wk / 15 sessions; 20 min Motor imagery-based brain-computer interface training CON: 3 wk / 15 sessions; 20 min Functional electric stimulation	MBI (score) Activities of daily living pre-/post-intervention	**MBI**: INT pre: 54.5 (15.41) CON pre: 52.5 (18.30) INT post: 80.0 (17.26) CON post: 62.5 (17.33)	+
Rizkalla [86]	INT (4 F, 5 M); 65.0 ± 9.31 yrs; right hemisphere; cognitive dysfunction CON (3 F, 6 M); 67.0 ± 7.81 yrs; right hemisphere; cognitive dysfunction	INT: 0.5 (0.1) months CON: 0.6 (0.1) months	INT (*n* = 9); visual skills training CON (*n* = 9); physical-, occupational- and speech therapy	INT: 4 wk / 20 sessions; 60 min Visuospatial scanning, visuospatial construction, complex visuospatial-visuomotor activity, mirror neuron training, complex visual skills homework CON: no details reported	MMSE (score) Global cognitive function MoCA (score) Global cognitive function TMT-A (sec) Visual processing speed TMT-B (sec) Cognitive flexibility MBI (score) Activities of daily living WAIS-DS (score) Working memory function pre-/post-intervention	**MMSE**: INT pre: 20.67 (2.35) CON pre: 19.00 (3.64) INT post: 26.00 (2.45) CON post: 19.22 (4.87) **MoCA**: INT pre: 17.11 (2.57) CON pre: 16.11 (3.33) INT post: 21.78 (2.99) CON post: 15.89 (6.11) **TMT-A**: INT pre: 145.00 (101.00) CON pre: 136.11 (100.62) INT post: 109.11 (90.81) CON post: 133.78 (101.60) **TMT-B**: INT pre: 257.22 (65.72) CON pre: 273.67 (64.14) INT post: 201.56 (77.36) CON post: 270.89 (66.31) **MBI**: INT pre: 34.44 (18.45) CON pre: 39.44 (8.08) INT post: 61.67 (24.75) CON post: 42.78 (7.95) **WAIS-DS**: INT pre: 5.11 (1.27) CON pre: 5.44 (1.13) INT post: 6.67 (1.41) CON post: 5.67 (1.66)	+
Tramontano et al. [87]	INT (6 F, 9 M); 52.8 ± 9.2 yrs; various; cognitive dysfunction CON (7 F, 8 M); 61.7 ± 15.2 yrs; various; cognitive dysfunction	< 6 months	INT (*n* = 15); visual-spatial training CON (*n* = 15); balance training combined with conventional rehabilitation	INT: 4 wk / 20 sessions; 60 min 40 min of conventional rehabilitation 20 min of visual-spatial, specular vision, and target identification training CON: 4 wk / 20 sessions; 60 min 40 min of conventional rehabilitation 20 min of balance training	MBI (score) Activities of daily living pre-/post-intervention	**MBI**: INT pre: 70.9 (18.71) CON pre: 69.1 (24.42) INT post: 92.4 (11.12) CON post: 70.1 (21.36)	+
Moon et al. [88]	INT (10 F, 10 M); 54.00 ± 14.63 yrs; NA; cognitive dysfunction CON (11 F, 9 M); 56.95 ± 12.86 yrs; NA; cognitive dysfunction	INT: 1.0 (0.1) months CON: 1.1 (0.1) months	INT (*n* = 18); eye-tracking visual cognitive training CON (*n* = 21); conventional cognitive training	INT: 6 wk / 30 sessions; 30 min Eye-tracking visual attention and processing training CON: 6 wk / 30 sessions; 30 min Computerized cognitive rehabilitation	MMSE (score) Global cognitive function MBI (score) Activities of daily living V-CPT (number) Selective attention pre-/post-intervention	**MMSE**: INT pre: 21.55 (5.68) CON pre: 21.50 (4.13) INT post: 25.70 (3.37) CON post: 25.10 (3.59) **MBI**: INT pre: 46.25 (17.80) CON pre: 59.00 (23.86) INT post: 62.75 (18.33) CON post: 76.05 (20.12) **V-CPT**: INT pre: 45.30 (20.16) CON pre: 39.85 (15.57) INT post: 53.00 (21.94) CON post: 42.90 (15.35)	-
Van Wyk [89]	INT (6 F, 6 M); 19–74 yrs; NA; visual disorders CON (6 F, 6 M); 19–74 yrs; NA; visual disorders	NA	INT (*n* = 12); eye movement training CON (*n* = 12); task-specific activities	INT: 4 wk / 20 sessions; 45 min Saccadic eye movement training and visual scanning with task-specific activities CON: 4 wk / 20 sessions; 45 min Task-specific activities	MMSE (score) Global cognitive function MBI (score) Activities of daily living pre-/post-intervention	**MMSE**: INT pre: 21.0 (3.95) CON pre: 20.7 (5.12) INT post: 25.4 (2.02) CON post: 24.1 (3.06) **MBI**: INT pre: 42.9 (18.40) CON pre: 46.3 (18.11) INT post: 85.4 (16.44) CON post: 65.4 (27.84)	+
Zhu et al. [90]	INT (6 F, 10 M); 57.75 ± 16.75 yrs; various; motor dysfunction CON (7 F, 8 M); 56.89 ± 17.93 yrs; various; motor dysfunction	< 3 months	INT (*n* = 16); visual feedback training CON (*n* = 15); conventional rehabilitation	INT: 8 wk / 48 sessions; 210–270 min 30-minute visual feedback training 180–240 min of conventional rehabilitation CON: 8 wk / 48 sessions; 180–240 min 180–240 min conventional rehabilitation, Bobath and Brunnstrom approaches, proprioceptive neuromuscular facilitation techniques, physical exercise, daily activities, physical- and occupational therapy	MBI (score) Activities of daily living pre-/post-intervention	**MBI**: INT pre: 42.75 (11.09) CON pre: 43.78 (12.11) INT post: 72.33 (11.82) CON post: 63.75 (10.45)	+
Zhang et al. [91]	INT (6 F, 14 M); 66.90 ± 4.42 yrs; NA; NA CON (5 F, 15 M); 67.55 ± 4.94 yrs; NA; NA	INT: 2.2 (0.3) months CON: 2.3 (0.3) months	INT (*n* = 20); visual feedback training CON (*n* = 20); balance training	INT: 3 wk / 15 sessions; 40 min 20 min of visual feedback training 20 min of balance training CON: 3 wk / 15 sessions; 20 min 20 min of balance training	MBI (score) Activities of daily living pre-/post-intervention	**MBI**: INT pre: 29.50 (8.87) CON pre: 30.00 (8.89) INT post: 61.75 (10.3) CON post: 49.50 (10.99)	+
Wang et al. [92]	INT (1 F, 14 M); 56.73 ± 10.73 yrs; NA; motor dysfunction CON (14 M); 59.43 ± 9.75 yrs; NA; motor dysfunction	INT: 4.0 (1.2) months CON: 4.7 (1.5) months	INT (*n* = 15); visuomotor training CON (*n* = 14); health education combined with conventional rehabilitation	INT: 4 wk / 20 sessions; 210 min 30-minute visuomotor training 180 min of conventional rehabilitation CON: 4 wk / 20 sessions; 210 min 30-minute health education 180 min of conventional rehabilitation	MBI (score) Activities of daily living pre-/post-intervention	**MBI**: INT pre: 57.73 (16.24) CON pre: 63.82 (16.25) INT post: 78.64 (13.73) CON post: 71.71 (17.94)	++
Braun et al. [93]	INT (13 F, 5 M); 77.7 ± 7.4 yrs; NA; NA CON (9 F, 9 M); 77.9 ± 7.4 yrs; NA; NA	INT: 1.4 (0.6) months CON: 1.1 (0.8) months	INT (*n* = 18); visuomotor training CON (*n* = 18); difficult task homework combined with conventional rehabilitation	INT: 6 wk; no details reported Visuomotor training combined with multi-professional rehabilitation CON: 6 wk; no details reported Multi-professional rehabilitation	MBI (score) Activities of daily living pre-/post-intervention	**MBI**: INT pre: 11.17 (4.1) CON pre: 12.22 (5.4) INT post: 15.00 (4.5) CON post: 14.94 (5.5)	+
Luukkainen-Markkula et al. [94]	INT (4 F, 2 M); 57.8 ± 11.8 yrs; right hemisphere; motor dysfunction CON (3 F, 3 M); 59.5 ± 8.4 yrs; right hemisphere; motor dysfunction	INT: 3.2 (2.1) months CON: 2.7 (2.2) months	INT (*n* = 6); visual scanning training CON (*n* = 6); arm activation training combined with conventional rehabilitation	INT: 8 wk / 32 sessions; 90 min 20 min of visual scanning training 30 min of physical therapy 40 min of occupational therapy CON: 8 wk / 32 sessions; 90 min 45 min of arm activation training 25 min of physical therapy 20 min of occupational therapy	WAIS-DS (score) Working memory function pre-/post-intervention	**WAIS-DS**: INT pre: 9.5 (3.7) CON pre: 8.4 (5.5) INT post: 12.0 (3.9) CON post: 10.6 (5.1)	+

INT = Intervention group; CON = Control group; MoCA = Montreal Cognitive Assessment; WAIS-DS = Wechsler Adult Intelligence Scale digit span score; MMSE = Mini-Mental Status Examination; MBI = Modified Barthel Index; V-CPT = Visual Continuous performance test; TMT-A = Trail Making Test Part A; TMT-B = Trail Making Test Part B; ST = Stroop test; Level of evidence: low quality (−), acceptable quality (+), and high quality (++), according to the Scottish Intercollegiate Guidelines Network Methodology checklist

### Participant characteristics

A total of 889 participants were examined in the included studies of this meta-analysis. All participants had a history of diagnosed stroke and were aged between 30 and 77 years. One study examined participants with a mean age of 30.9–39.8 years [56], 14 studies of 52.5–62.6 years [55, 58, 59, 76, 78, 80, 82–84, 87, 88, 90, 92, 94], six of 64.0–67.6 years [57, 77, 81, 85, 86, 91], and three of 70.7–77.7 years [79, 89, 93]. A total of 357 women and 532 men were studied. One study considered one woman and 28 men [92] and another five women and 25 men [76]. All other studies had a more balanced female-to-male ratio. The localisation was indicated by five studies with right hemisphere [58, 78, 81, 86, 94], six with various [55, 57, 59, 77, 87, 90], while all others did not provide any information. The main symptoms of the participants indicated eleven studies with cognitive dysfunction [55–57, 59, 78, 79, 82, 83, 86–88], five with motor dysfunction [58, 77, 90, 92, 94], three with visual disorders [80, 81, 89], and five did not provide any information [76, 84, 85, 91, 93]. For the time since stroke event, one study gave no indication [89], two generally less than 3 months [55, 90], two from 3 to 6 months [56, 80], three less than 6 months [57, 59, 87], two 0.5–0.6 months [58, 86], seven 1.0–3.2 months [77, 81, 85, 88, 91, 93, 94], two 4.0–4.9 months [79, 92], one more than 6 months [84], and four 8.3–20.8 months [76, 78, 82, 83]. Six populations were analyzed in China [55, 57, 58, 90–92], four in Korea [79, 83, 84, 88], two in Germany [77, 81], two in Italy [56, 87], two in Canada [85, 86], and one each in Pakistan [80], Egypt [76], Iran [78], the Netherlands [93], Finland [94], Russia [59], South Africa [89], and Sweden [82].

### Intervention characteristics

In the selection of therapy methods, four studies illustrated general visual skills training [55, 56, 85, 86], four visuomotor training [76, 79, 92, 93], four visual-spatial training [59, 82, 83, 87], three visual feedback training [58, 90, 91], three visual scanning training [80, 81, 94], two eye-movement training [57, 89], and two visual-cognitive training [77, 78]. One study each used vision control dual-task training [84] and eye-tracking visual cognitive training [88] as an intervention approach. All included studies differed significantly in the total period of intervention, number of sessions, and session duration, ranging from 10 days to 10 weeks, eight to 48 sessions, and 20 to 270 min.

Six studies carried out eight to 16 sessions with a total of 300–600 min of visual training [55, 58, 59, 77, 78, 91], 10 conducted 20–25 sessions and 600–4200 min [80–87, 89, 92], and six 28–48 sessions and 840-11520 min [57, 76, 79, 88, 90, 94]. One study did not provide information on the number of sessions or session duration [93] and one did not provide information on session duration [56].

### Outcome measures

Of the included studies, nine measured at least one outcome for cognitive function [55, 59, 76, 78, 82–85, 94], nine only measured activities of daily living [58, 77, 80, 81, 87, 90–93], and six studies considered both outcomes [56, 57, 79, 86, 88, 89]. In the area of cognitive function, six examined global cognitive function [55–57, 59, 76, 89], six executive functions [78, 82–85, 94], and three considered both outcomes [79, 86, 88]. To measure global cognitive function the MoCA was used in three studies [55, 57, 76], the MMSE in four [56, 79, 88, 89], and two studies considered both tests [59, 86]. The WAIS-DS was used to measure working memory function in five studies [79, 82, 83, 86, 94], the TMT-A for visual processing time in five [79, 83–86], and the TMT-B for cognitive flexibility in three studies [84–86]. The V-CPT was used as a measurement instrument for selective attention in three studies [78, 79, 88] and the ST in two, one of which used the ST to measure selective attention [84] and the other to measure inhibitory control [82]. Table 3 shows all the effects of the visual skills interventions on cognitive and executive function as well as activities of daily living in stroke patients.

### Effects of visual skills training on global cognitive function, executive functions and activities of daily living in stroke patients

**Fig. 2:**
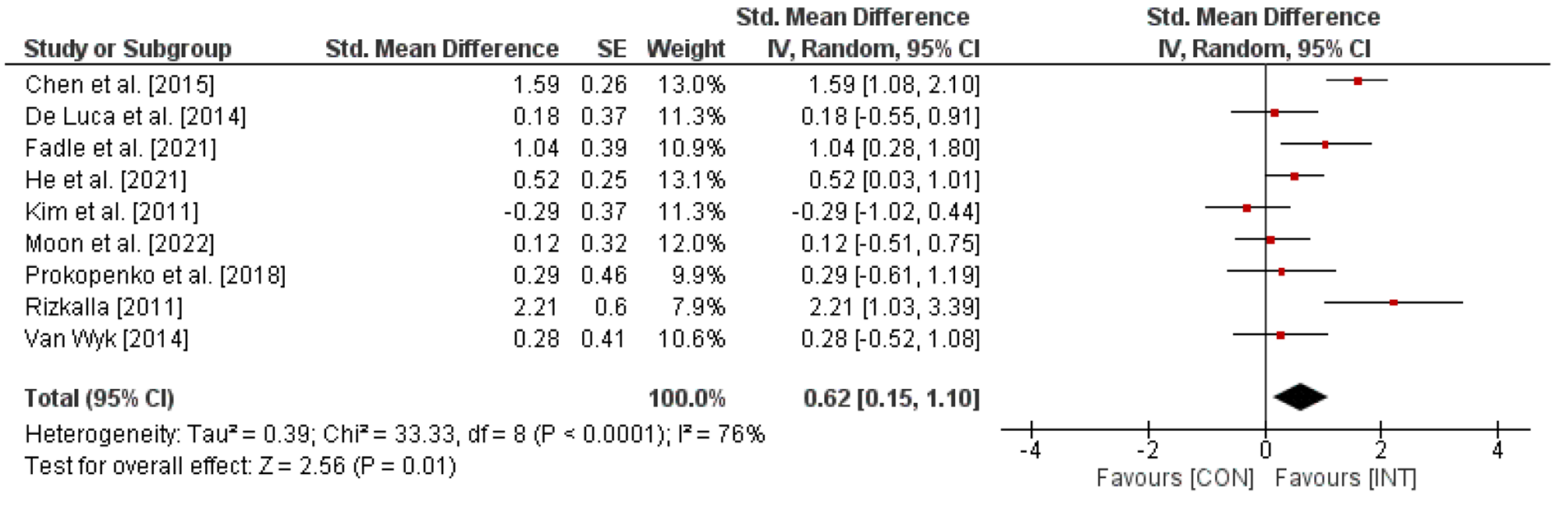
shows the effects of visual skills training on global cognitive function. In total, the weighted mean *SMD* resulted in 0.62 (*Chi*^2^ = 33.33, *df* = 8, *p* < .0001, *I*^2^ = 76%), indicating a moderate-sized effect favoring the INT groups

Figure 2. Effects of visual skills training on global cognitive function (e.g., MMSE) in stroke patients. *CI* = confidence interval; *CON* = control group; *df =* degrees of freedom; *INT* = intervention group; *IV* = inverse variance; *SE* = standard error; *Std.* = standard.

**Fig. 3:**
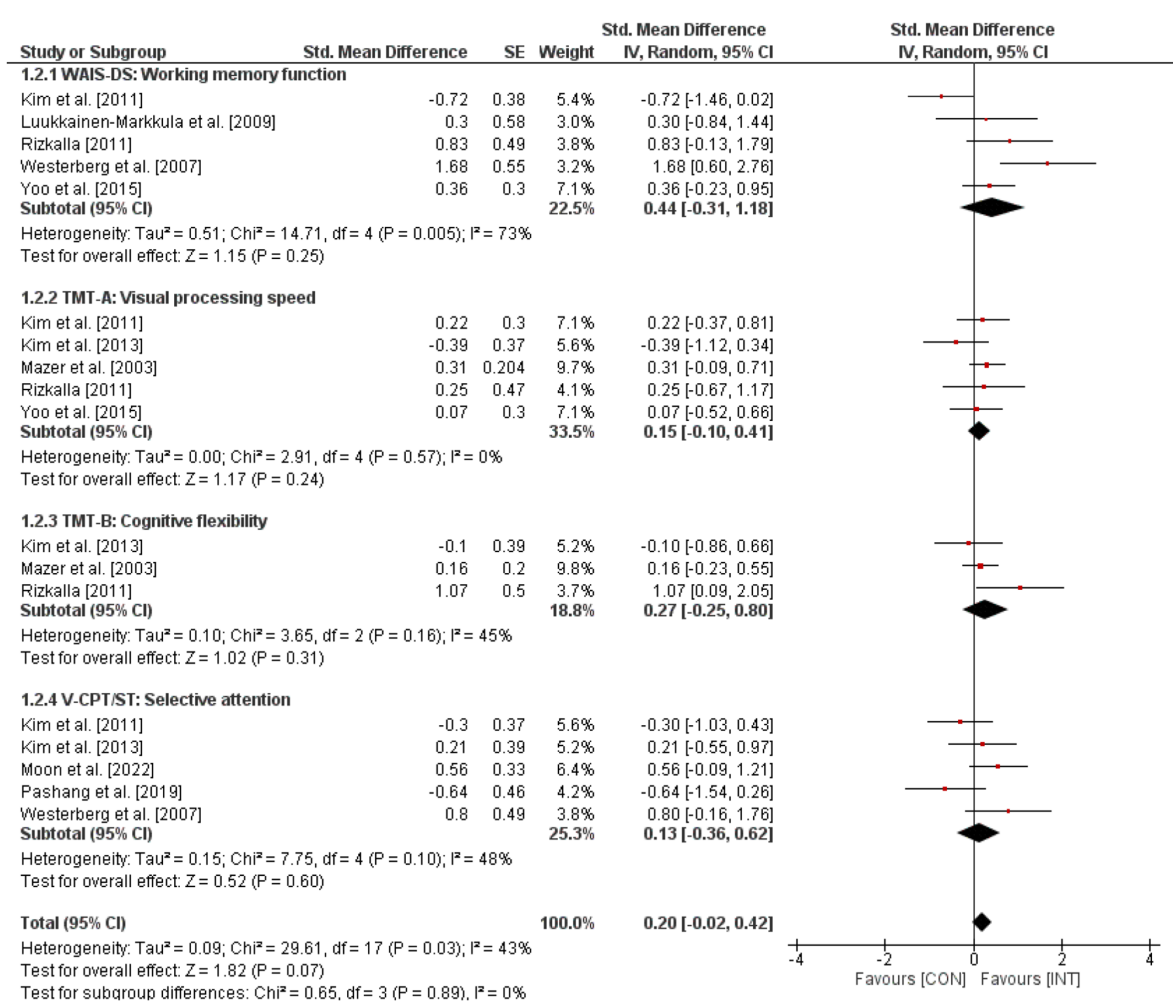
Effects of visual skills training on executive functions (e.g., WSAI-DS) in stroke patients. *CI* = confidence interval; *CON* = control group; *df =* degrees of freedom; *INT* = intervention group; *IV* = inverse variance; *SE* = standard error; *Std.* = standard

The effects of visual skills training on executive functions in stroke patients are outlined in Fig. 3. The weighted mean *SMD* amounted to 0.20 (*Chi*^2^ = 29.61, *df* = 17, *p* = .03, *I*^2^ = 43%), which indicates a small-sized effect in favor of the INT groups. Further, a subgroup-analyses revealed small-sized effects for working memory function (*SMD* = 0.44, *Chi*^2^ = 14.71, *df* = 4, *p* = .005, *I*^2^ = 73%), visual processing speed (*SMD* = 0.15, *Chi*^2^ = 2.91, *df* = 4, *p* = .57, *I*^2^ = 0%), cognitive flexibility (*SMD* = 0.27, *Chi*^2^ = 3.65, *df* = 2, *p* = .16, *I*^2^ = 45%), and selective attention (*SMD* = 0.13, *Chi*^2^ = 7.75, *df* = 4, *p* = .10, *I*^2^ = 48%).

**Fig. 4:**
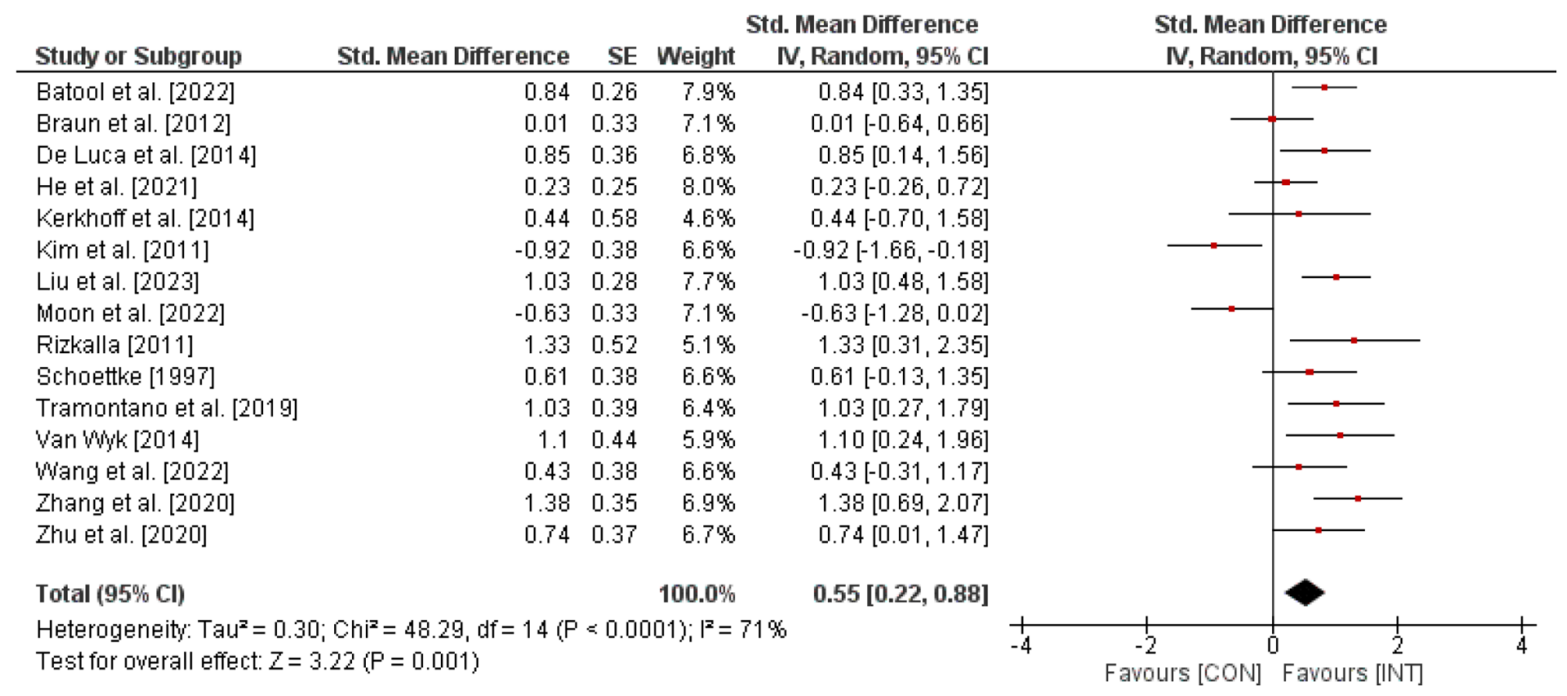
Effects of visual skills training on activities of daily living (e.g., MBI) in stroke patients. *CI* = confidence interval; *CON* = control group; *df =* degrees of freedom; *INT* = intervention group; *IV* = inverse variance; *SE* = standard error; *Std.* = standard

For the effects of visual skills training on activities of daily living in stroke patients, illustrated in Fig. 4, the weighted mean SMD indicated 0.55 (*Chi*^2^ = 48.29, *df* = 14, *p* < .0001, *I*^2^ = 71%), thus revealing a moderate effect in favor of the INT-groups.

### Reporting bias and sensitivity analysis

**Fig. 5:**
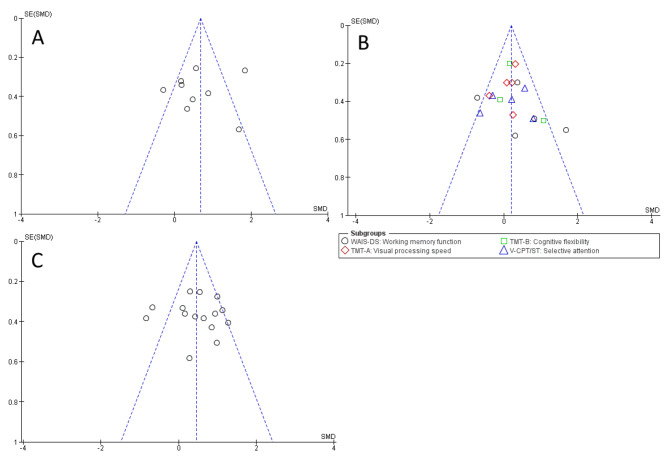
Funnel plots for publication bias assessment regarding (**A**) global cognitive function, (**B**) executive functions, and (**C**) activities of daily living. *SE*: standard error; *SMD*: standardized mean difference

Funnel Plots are illustrated in Fig. 5A-C. For all measures, the symmetry is limited, indicating a possible publication bias. However, Egger’s test showed no asymmetry for global cognitive function (value = -0.374; *p* = .709), executive functions (value = 1.058; *p* = .290), and activities of daily living (value = 0.565; *p* = .572), respectively. The results therefore give no indication of a publication bias.

After finding high heterogeneity (I^2^ = 76%) regarding global cognitive function, the study by Chen et al. [55] was removed for sensitivity analysis, resulting in a reduced I^2^ value of 58%. Further removing the study by Rizkalla [86] reduced heterogeneity significantly (I^2^ = 18%), also revealing a reduction of the weighted mean *SMD* to 0.32, indicating small-sized effects. An sub-group analysis was conducted to explore the potential mediating effect of time since stroke on global cognitive function scores. The analysis revealed a statistically significant association (*p* = .027, Table 4) between time since stroke and global cognitive function, suggesting that time since stroke may serve as a moderating factor.

**Table 4:** Sub-group/moderator analysis of time since stroke and global cognitive function

Mixed-Effects Model (k = 8)						
	Estimate	se	Z	*p*	CI Lower Bound	CI Upper Bound
Intercept	1.748	0.506	3.45	< 0.001	0.756	2.740
Moderator	-0.496	0.225	-2.21	0.027	-0.936	-0.056

Note. Tau² Estimator: Maximum-Likelihood; Van Wyk [89] did not report any data and was therefore excluded

As a high heterogeneity was found for executive functions in the working memory function subgroup (I^2^ = 73%), the study by Kim et al. [79] was removed for the sensitivity analysis, which reduced I^2^ to a value of 39%. The exclusion of Westerberg et al. [82] resulted in a homogeneous outcome (I^2^ = 0%) and did not alter the effect size (*SMD* = 0.46). When looking at the study characteristics, the large number of men compared to women was apparent in both studies. A quotient was calculated to determine the proportion of male participants in each study and analyzed as a potential moderator variable. The analysis showed no significant correlation (*p* = .420) between the average number of male participants and the working memory function (Table 5).

**Table 5:** Sub-group/moderator analysis of proportion of male participants and working memory function

Mixed-Effects Model (k = 5)						
	Estimate	se	Z	*p*	CI Lower Bound	CI Upper Bound
Intercept	-0.794	1.54	-0.515	0.607	-3.815	2.228
Moderator	2.344	2.90	0.807	0.420	-3.349	8.037

Note. Tau² Estimator: Maximum-Likelihood

## Discussion

To the best of the authors’ knowledge, this is the first systematic review with meta-analysis on the effects of visual skills training on cognitive functions in stroke patients and provides a quantitative analysis. Despite the influence of the visual system on the outcomes of a variety of post-stroke rehabilitation strategies, the empirical knowledge on the impact of interventions explicitly targeting visual skills is deficient. The meta-analysis conducted includes 24 studies and illustrates (i) moderate-sized effects on global cognitive function and (ii) small-sized effects on executive functions and activities of daily living in favor of the INT groups. However, due to the high heterogeneity and moderating variables, the results are not very robust and must therefore be interpreted with caution.

### Effects of visual skills training on measures of global cognitive function

The included studies that investigated the effects of visual skills training on global cognitive function predominantly showed positive effects. In terms of the intervention approach, the studies illustrated mixed results. Studies involving general visual skills training were the only ones to exhibit large effects [55, 86], while another study showed low effects [56]. However, the latter was the only one of this group to combine visual skills training with conventional rehabilitation and did not indicate the amount of visual skills training. The age of the participants, the time since the stroke, the total length of the intervention, the number of sessions, and their duration did not appear to influence the outcomes observed in the studies. Studies in which visual skills training explicitly involved the loading of multiple cognitive components and systems [55, 86] illustrated stronger effects than those in which the intervention was less complex [56, 57, 59, 76, 79, 88, 89]. This is consistent with the findings of Mathews [42] indicating that increased cognitive load due to higher complexity enhances cortical adaptation processes. This is also supported by the findings of Appelbaum and Erickson [95] who reported improved effectiveness of visual skills training in athletes by adding dual-tasks. The analysis revealed that training in visual skills had a significant impact on patients within three months of a stroke. However, small effects were observed beyond this period. These results are consistent with Bergsma et al. [96], who found that training the visual system has a stronger impact on cognitive function, particularly in the immediate post-stroke phase. This effect is more pronounced than in later stages. Recent research has shown that surviving neurons in the peri-infarcted tissue enlarge their dendritic trees and sprout axons, highlighting the importance of neuroplasticity, especially in the early phase [97]. In summary, visual skills training can improve global cognitive function, especially when it is more complex, involves additional cognitive load and is applied early after stroke. Future research should investigate visual skills interventions with and without additional cognitive load, as well as the type of cognitive load (e.g., visual, auditory, tactile).

### Effects of visual skills training on measures of executive function

The results of the studies that examined the effects on executive functions only partially illustrated positive effects. Among the subgroups, the greatest effects were observed in working memory function and cognitive flexibility. Correlations between the improvement of visual skills and certain subdomains of cognitive functions have been scarcely explored in the literature. Only Knöllner et al. [98] investigated the connection between visual skills and executive functions and referred to the strongest correlation between visual skills and working memory function. Regarding time since stroke, the studies with the longest time since stroke showed negative and the lowest positive effects for visual processing speed [84, 90] and negative effects for cognitive flexibility [84]. These results are in line with van de Ven et al. [99], who found that cognitive flexibility improves independently with increasing time after onset, even without training. This could limit the effects of therapy depending on how long ago the stroke occurred. When classifying the analysis on selective attention, it should be noted that the two studies with negative effects [78, 79] both had low methodological study quality and significantly lower values at baseline in the INT compared to the CON groups, so that a comparison of the treatment effects should be interpreted with caution. Overall, the heterogeneity of the included studies in terms of different tests and interventions is too high to draw therapy-relevant conclusions. It can be stated that the basic correlation between visual skills and executive functions should be investigated in future studies to prioritize interventions according to the individual limitations of stroke patients.

### Effects of visual skills training on measures of activities of daily living

The results in the area of activities of daily living as a secondary outcome of this meta-analysis were predominantly positive. It should be noted that the only two studies with negative effects [79, 88] were both of low study quality and the baseline values were significantly lower in the INT group than in the CON group. Even though the heterogeneity of the included studies is very high, it can be concluded that visual skills training to improve activities of daily living can be useful, taking into account the aforementioned aspects.

### Strengths and limitations of this systematic review with meta-analysis

There are obvious strengths and limitations to this article. One important aspect is that this is the first meta-analysis to evaluate the benefits of all visual system-based interventions in stroke patients and includes eight papers published in the last three years. Overall, the included studies are of low to high methodological quality. The most common methodological shortcomings are the lack of blinding of investigators and subjects or inadequate concealment methods. However, no study of unacceptable quality had to be excluded from the meta-analysis. Another weakness of our meta-analysis is the inconsistency of the studies regarding the interventions (different visual training methods, different control interventions, different intervention duration) and the heterogeneous location of the stroke event. Furthermore, the population of the included studies showed a broad spectrum of cognitive and motor symptoms, as well as partial impairment of the visual system. This means that only general, but no specific conclusions can be drawn about the effectiveness of the interventions. This must be taken into account when analyzing the results. The interpretation of the results is also limited by the small sample sizes of the included studies, with the largest being 97 participants, and by the fact that only a few studies investigated the long-term effects.

## Conclusions

The present systematic review with meta-analysis aimed to identify the existing evidence for training that explicitly focuses on visual skills in relation to cognitive and executive functions and also to establish a link with activities of daily living. The results presented in this meta-analysis suggest a potential relationship between visual skills training and the improvement of cognitive functions. Based on the analysis of this work, it can be stated that visual skills training, especially when designed to include a high cognitive load, can achieve considerable effects in the area of global cognitive function and should therefore be used in clinical practice. However, the large number of different interventions and variations in delivery in this area make it difficult to draw precise conclusions about the nature of the intervention, so further research aimed at specifying and refining the approaches used in visual skills training appears necessary. Furthermore, the clinical manifestations of stroke are non-specific and highly variable, as different functional systems can be affected. In future research, therefore, not only the brain areas of the lesion should be named, but in particular the functional limitations of the affected individual should be identified. This approach would allow for a more precise understanding of the therapy outcomes, attributing them more accurately to the specific functional impairments.

### Electronic supplementary material

Below is the link to the electronic supplementary material.

Supplementary Material 1

Supplementary Material 2

## Data Availability

No datasets were generated or analysed during the current study.
