# Supplementary material for: The effects of visual skills training on cognitive and executive functions in stroke patients: a systematic review with meta-analysis
Source: J Neuroeng Rehabil. 2024 Mar 26;21:41. doi: 10.1186/s12984-024-01338-5 (PMC10967170; doi:10.1186/s12984-024-01338-5)
Supplement: Supplementary file 2 — Supplementary Material 2 [file 12984_2024_1338_MOESM2_ESM.docx]

|  | Chen et al. (2015) | Fadle et al. (2021) | Schoettke (1997) | Pashang et al. (2019) | He et al. (2021) | Kim et al. (2011) | Batool et al. (2022) | De Luca et al. (2014) | Kerkhoff et al. (2014) | Westerberg et al. (2007) | Prokopenko et al. (2018) | Yoo et al. (2008) | Kim et al. (2013) | Mazer et al. (2003) | Liu et al. (2023) | Rizkalla (2008) | Tramontano et al. (2019) | Moon et al. (2022) | Van Wyk (2014) | Zhu et al. (2020) | Zhang et al. (2020) | Wang et al. (2022) | Braun et al. (2012) | Luukkainen-Markkula et al. (2009) |
| --- | --- | --- | --- | --- | --- | --- | --- | --- | --- | --- | --- | --- | --- | --- | --- | --- | --- | --- | --- | --- | --- | --- | --- | --- |
| 1.1 The study addresses an appropriate and clearly focused question. | Yes | Yes | Yes | Yes | Yes | Yes | Yes | Yes | Yes | Yes | Yes | Yes | Yes | Yes | Yes | Yes | Yes | Yes | Yes | Yes | Yes | Yes | Yes | Yes |
| 1.2 The assignment of subjects to treatment groups is randomised. | Yes | Yes | Yes | Yes | Yes | Yes | Yes | Yes | Yes | Yes | Yes | Yes | Yes | Yes | Yes | Yes | Yes | No | Yes | Yes | Yes | Yes | Yes | Yes |
| 1.3 An adequate concealment method is used. | No | No | No | No | Yes | No | Yes | No | Yes | Yes | No | No | No | Yes | Yes | No | No | No | No | No | Yes | Yes | Yes | Yes |
| 1.4 The design keeps subjects and investigators ‘blind’ about treatment allocation. | No | No | No | No | Yes | No | Yes | No | No | No | No | No | No | No | No | No | No | No | Yes | Yes | No | Yes | No | No |
| 1.5 The treatment and control groups are similar at the start of the trial. | Yes | Yes | Yes | No | Yes | No | Yes | No | Yes | Yes | No | Yes | Yes | Yes | Yes | Yes | Yes | Yes | Yes | Yes | Yes | Yes | Yes | Yes |
| 1.6 The only difference between groups is the treatment under investigation. | Yes | Cs | Yes | No | Yes | No | Yes | No | Yes | No | Yes | Yes | Yes | Yes | Yes | Yes | Yes | Yes | Yes | Yes | Yes | Yes | Yes | No |
| 1.7 All relevant outcomes are measured in a standard, valid and reliable way. | Yes | Yes | Yes | Yes | Yes | Yes | Yes | No | Yes | Yes | No | Yes | Yes | Yes | Yes | Yes | Yes | Yes | Yes | Yes | Yes | Yes | Yes | Yes |
| 1.8 What percentage of the individuals or clusters recruited into each treatment arm of the study dropped out before the study was completed? | 0% | 0% | 0% | 0% | 0% | Cs | 0% | 0% | 0% | 14% | 0% | 0% | 0% | 13% | 7% | 0% | 0% | Cs | 0% | 33% | 0% | 26% | 6% | 0% |
| 1.9 All the subjects are analysed in the groups to which they were randomly allocated (often referred to as intention to treat analysis). | Yes | Yes | Yes | Yes | Yes | Yes | Yes | Yes | Yes | Yes | Yes | Yes | Yes | Yes | Yes | Yes | Yes | Yes | Yes | Yes | Yes | Yes | Yes | Yes |
| 1.10 Where the study is carried out at more than one site, results are comparable for all sites. | Na | Na | Na | Na | Na | Na | Na | Na | Na | Na | Na | Na | Na | Na | Na | Na | Na | Na | Na | Na | Na | Na | Yes | Na |
| **2.1** How well was the study done to minimise bias?  Code as follows: High quality (++), Acceptable quality (+), Low quality (-) | + | - | + | - | ++ | - | ++ | - | + | + | - | + | + | ++ | + | + | + | - | + | + | + | ++ | + | + |
| 2.2 Taking into account clinical considerations, your evaluation of the methodology used, and the statistical power of the study, are you certain that the overall effect is due to the study intervention? | Yes | Yes | Yes | Yes | Yes | Yes | Yes | Yes | Yes | Yes | Yes | Yes | Yes | Yes | Yes | Yes | Yes | Yes | Yes | Yes | Yes | Yes | Yes | Yes |
| 2.3 Are the results of this study directly applicable to the patient group targeted by this guideline? | Yes | Yes | Yes | Yes | Yes | Yes | Yes | Yes | Yes | Yes | Yes | Yes | Yes | Yes | Yes | Yes | Yes | Yes | Yes | Yes | Yes | Yes | Yes | Yes |
